# Low-Power Phototransistor with Enhanced Visible-Light Photoresponse and Electrical Performances Using an IGZO/IZO Heterostructure

**DOI:** 10.3390/ma17030677

**Published:** 2024-01-30

**Authors:** Yu Bin Kim, Jun Hyung Jeong, Min Ho Park, Jung Min Yun, Jin Hyun Ma, Hyoun Ji Ha, Seong Jae Kang, Seong Jun Kang

**Affiliations:** 1 Department of Advanced Materials Engineering for Information and Electronics, Kyung Hee University, Yongin 17104, Republic of Korea; bebeto@khu.ac.kr (Y.B.K.); 2016101120@khu.ac.kr (J.H.J.); triolight@khu.ac.kr (M.H.P.); msd426@khu.ac.kr (J.M.Y.); majh0506@khu.ac.kr (J.H.M.); localha99@khu.ac.kr (H.J.H.); andrew970728@khu.ac.kr (S.J.K.); 2 Integrated Education Program for Frontier Materials (BK21 Four), Kyung Hee University, Yongin 17104, Republic of Korea

**Keywords:** oxide–oxide heterostructure, low power consumption, visible-light phototransistor, defect states, optoelectronics, solution process

## Abstract

In this study, we demonstrated the effective separation of charge carriers within the IGZO/IZO heterostructure by incorporating IZO. We have chosen IGZO for its high mobility and excellent on–off switching behavior in the front channel of our oxide–oxide heterostructure. Similarly, for an additional oxide layer, we have selected IZO due to its outstanding electrical properties. The optimized optoelectronic characteristics of the IGZO/IZO phototransistors were identified by adjusting the ratio of In:Zn in the IZO layer. As a result, the most remarkable traits were observed at the ratio of In:Zn = 8:2. Compared to the IGZO single-layer phototransistor, the IGZO/IZO(8:2) phototransistor showed improved photoresponse characteristics, with photosensitivity and photoresponsivity values of 1.00 × 10^7^ and 89.1 AW^−1^, respectively, under visible light wavelength illumination. Moreover, the electrical characteristics of the IGZO/IZO(8:2) transistor, such as field effect mobility (*μ_sat_*) and current on/off ratio (*I_on_*/*I_off_*), were highly enhanced compared to the IGZO transistor. The *μ_sat_* and *I_on_*/*I_off_* were increased by about 2.1 times and 2.3 times, respectively, compared to the IGZO transistor. This work provides an approach for fabricating visible-light phototransistors with elevated optoelectronic properties and low power consumption based on an oxide–oxide heterostructure. The phototransistor with improved performance can be applied to applications such as color-selective visible-light image sensors and biometric sensors interacting with human–machine interfaces.

## 1. Introduction

In recent times, with the growing importance of electronic industries including Internet of Things (IoT) technologies, healthcare systems, light communications, and autonomous mobile systems, the crucial role of photodetectors in connecting users and surrounding objects has gained increased prominence [1]. In particular, the demand for obtaining visible-light photodetectors with high performance and low power consumption is crucial for their use in numerous industries, such as biomedical health monitoring systems, electronic eyes, visible light communication (VLC), and advanced driver assistance systems (ADAS) [2,3,4]. Among numerous architectures of photodetectors, such as photodiodes, photoresistors, and phototransistors, the phototransistor structure provides numerous advantages for high-performance photodetectors, such as low off-state current and facile adjustable photocurrent by drain and gate bias. Therefore, to achieve efficient information processing in the IoT and innovative applications such as wearable sensors and integrated devices, photodetectors based on the transistor structure have been widely investigated to enhance the performance of devices, including photoresponsivity and reliability [5,6,7].

For obtaining high-performance phototransistors, metal oxide semiconductors, such as zinc oxide (ZnO) and indium-gallium-zinc-oxide (IGZO) have been used as the channel materials, owing to their excellent characteristics including high field effect mobility (*μ_sat_*), low off-current, and high current on/off ratio (*I_on_*/*I_off_*) [8,9,10]. However, due to their wide band gap characteristics, the detection range of these materials is primarily restricted to the ultraviolet (UV) region, owing to the insufficient photon energy of visible light wavelengths to excite the valence electrons of these oxide semiconductors into the conduction band [10,11]. 

To overcome these limitations, research has been conducted on heterostructure phototransistors utilizing narrow band gap materials as an additional layer, such as two-dimensional (2D) materials, quantum dots (QDs), perovskites, and organic compounds. However, their intrinsically low electrical characteristics and unfavorable uniformity with oxide result in diminished optoelectronic properties of the device, deteriorating its capability to generate photocurrent and its response time [12,13,14,15,16,17]. Moreover, the ligand of QDs can impede carrier transport, and the narrow band gap materials are vulnerable to ambient air and moisture [18,19,20,21,22]. 

Therefore, in recent investigations, the emphasis has been on utilizing the defect states of oxide semiconductors, such as oxygen vacancies, to widen the detection range from UV to visible range [23,24,25,26]. Numerous methods such as doping, mechanochemical treatments, and forming a heterostructure with metal oxides have been studied to enhance visible-light photoresponse capability [26,27,28]. Methods such as doping or mechanochemical treatments can generate a large amount of oxygen vacancies, resulting in improved characteristics for detecting visible light. However, since the efficient separation of photo-generated electron–hole pairs should be considered for obtaining a much higher amount of photocurrent, research on hetero-structured phototransistors has been widely investigated.

Numerous oxide materials, such as TiO_2_, ZnON, and SnO, have been adopted for the oxide–oxide heterostructure; however, these materials also reduce the optoelectronic characteristics of the device, owing to their intrinsically low electrical characteristics [23,29,30]. This prevents the effective transfer of photo-generated charge carriers and deteriorates the optoelectronic characteristics. 

Here, we have selected IGZO as the front channel and indium zinc oxide (IZO) as the back channel in the oxide–oxide heterostructure. This decision was based on the superior electrical properties exhibited by IZO compared to the back-channel oxides studied in previous research, aiming to mitigate the decline in electrical performance [31]. By optimizing the In:Zn ratio in IZO, we identified the IGZO/IZO transistor with superior optoelectrical performance. We prepared three types of IZO solutions by varying the In:Zn ratios in IZO. The optimized device was the IGZO/IZO(8:2) phototransistor, which exhibited photosensitivity and photoresponsivity values of 1.00 × 10^7^ and 89.1 AW^−1^ under 520 nm wavelength light, respectively, which were significantly higher values compared to the single-layered IGZO device. Furthermore, electrical characteristics such as *μ_sat_* and *I_on_*/*I_off_* were also improved compared to the IGZO single-layered transistor. The device showed visible-light photoresponse characteristics with a low power consumption of 4.98 pJ and 0.357 nJ under periodic 520 and 450 nm wavelength light pulses, respectively. Our results suggest useful methods for fabricating solution-processable oxide-based visible-light phototransistors.

## 2. Materials and Method

### 2.1. Solution Synthesis

The IGZO solution was composed of indium nitrate hydrate (In(NO_3_)_2_·xH_2_O, Sigma Aldrich), gallium nitrate hydrate (Ga(NO_3_)_2_·xH_2_O, Sigma Aldrich, St. Louis, MO, USA), and zinc nitrate hexahydrate (Zn(NO_3_)_2_·6H_2_O, Sigma Aldrich) with a molar ratio of 6:1:2, dissolved in ethylene glycol monomethyl ether (Daejung, Siheung, Republic of Korea). The IZO solution was synthesized by blending In(NO_3_)_2_·xH_2_O and Zn(NO_3_)_2_·6H_2_O with ethylene glycol monomethyl ether (Daejung). For the division of ratios, IZO solutions were prepared by mixing the In precursor solution and Zn precursor solution in proportions of 8:2, 5:5, and 2:8. These processes resulted in a 0.1 M solution of IGZO and a 0.1 M solution of IZO. After stirring, they were stored in a humidity desiccator for more than a day before usage. 

### 2.2. Fabrication of the Device

A wafer with highly boron-doped Si, where SiO_2_ was thermally grown to a thickness of 100 nm, was successively ultrasonicated with deionized water (DI), acetone, and isopropyl alcohol (IPA) for 15 min, respectively. Subsequently, the wafer was subjected to UV–ozone treatment for 15 minutes to achieve a hydrophilic surface and remove any remaining organic residues, followed by two cycles of spin coating and annealing. First, the IGZO solution was spin-coated onto the substrate at 4000 rpm for 30 s and annealed at 120 °C for 5 min. The same spin-coating procedure was repeated, followed by annealing at 350 °C for 1 h. The IZO solutions were spin-coated onto the IGZO film at 5000 rpm for 30 s and annealed at 280 °C for 1 h. All the spin coating and annealing processes proceeded under an ambient atmosphere. To form the source/drain electrodes, 100 nm of aluminum was deposited on the substrate through thermal evaporation under 5 × 10^−6^ torr at a deposition rate of 3 Å/s. The channel length (L) and width (W) of 100 and 1000 μm, respectively, were defined using a metal shadow mask.

### 2.3. Characterization and Measurement of Films and Devices

The optoelectronic features of the device were explored using a semiconductor parameter analyzer (HP 4145B, Center for Detectors, Rochester, NY, USA) and a probe station, with an incident light power of around 4.5 mW cm^−2^. Measurements were performed under dark conditions and under illumination with light at wavelengths (λ) of 405, 450, 520, and 635 nm. The transmittance of the films was measured by a UV–visible spectrometer (Cary100, Agilent, Santa Clara, CA, USA). The device structure was analyzed by carrying out cross-sectional HR-TEM measurements using a JEM-2100F system (JEOL Ltd., Tokyo, Japan). Surface topologies and the RMS values of films were investigated by AFM (Dimension 3100 SPM, Digital Instruments, Tonawanda, NY, USA). XPS and UPS analysis (Thermo Fisher, NEXSA, Waltham, MA, USA) were conducted to investigate the interfacial properties with Al Kα (1486.8 eV) and He–I line (21.22 eV) sources, respectively.

## 3. Results and Discussion

**Figure 1:**
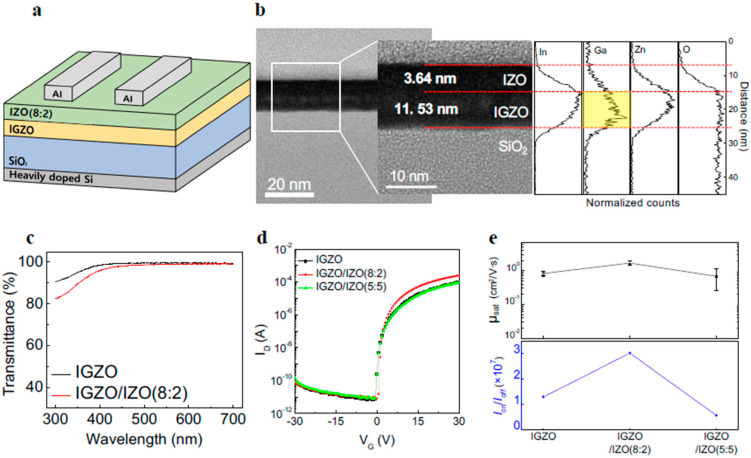
(**a**) Schematic illustration of the IGZO/IZO(8:2) phototransistor. (**b**) Cross-sectional HR-TEM image and EDS line scan data of the IGZO/IZO(8:2) film. (**c**) Optical transmittance spectra of the IGZO and IGZO/IZO(8:2) films. (**d**) The transfer curves of the IGZO, IGZO/IZO(8:2), and IGZO/IZO(5:5) phototransistors under dark state. (**e**) The error bars of field effect mobility and the on/off current ratio for IGZO, IGZO/IZO(8:2), and IGZO/IZO(5:5) devices.

Figure 1a displays the structure of the optimized phototransistor with an IGZO/IZO(8:2) heterostructure. Figure 1b depicts the cross-sectional HR-TEM image of the IGZO/IZO(8:2) film along with its corresponding EDS line spectra. The TEM image allows for the distinction between the IZO and IGZO layers, and the thickness of each layer was determined by the existence of Ga. The thickness of the IGZO layer containing Ga was approximately 11.53 nm, while the IZO layer without Ga was measured to be 3.64 nm, further confirmed by the EDS mapping image, as shown in Figure S1. Figure 1c shows the transmittance spectra of the IGZO and IGZO/IZO(8:2) films. Regardless of the structures of the films, all the films showed high transmittance over 95% in the wavelength range of 400 to 700 nm, indicating that the films can be used as transparent optoelectronic devices. To figure out whether the IZO layer above the IGZO layer directly affected the electrical characteristics, atomic force microscopy (AFM) was measured. Figure S2a–d shows the surface morphology and 3D topography of the IGZO, IGZO/IZO(8:2), IGZO/IZO(5:5), and IGZO/IZO(2:8) films, respectively, along with the root mean square (RMS) values of the surface roughness of the films. The RMS values of the films increased as the ratio of Zn in IZO increased, which might be owing to the surface properties of ZnO, where surface roughness rises with higher annealing temperatures and times [32,33]. It has also been widely reported that the surface roughness of In_2_O_3_ is not strictly affected by annealing temperature compared to ZnO [34,35]. Thus, a relatively high RMS value of 0.505 nm for the surface roughness of the IGZO/IZO(2:8) film might be attributed to the increased ratio of Zn in the IZO layer. This high surface roughness could negatively affect the overall performance of the phototransistor, including its electrical characteristics and stability [36]. Figure 1d depicts the transfer curves of the IGZO, IGZO/IZO(8:2), and IGZO/IZO(5:5) phototransistors under dark conditions. When the IZO(8:2) was stacked as an additional layer, the on-current increased compared to the IGZO single-layer device. Figure S3 shows the transfer curves of the IGZO/IZO(10:0), IGZO/IZO(2:8), and IGZO/IZO(0:10) phototransistors measured under dark conditions. The transfer curve of the IGZO/IZO(10:0) device exhibited conductive behavior, while the transfer curves of the IGZO/IZO(2:8) and IGZO/IZO(0:10) devices with high Zn showed deteriorated electrical characteristics. In addition, the transfer curves of IZO(8:2), IZO(5:5), and IZO(2:8) single-layer phototransistors are shown in Figure S4, revealing that the IZO single-layer devices also exhibit poor electrical characteristics. Figure 1e illustrates error bars denoting the standard deviations of *μ_sat_*, and the *I_on_*/*I_off_* for the IGZO, IGZO/IZO(8:2), and IGZO/IZO(5:5) phototransistors. Among the devices, the IGZO/IZO(8:2) transistor exhibited the highest electrical characteristics, such as *μ_sat_* and *I_on_*/*I_off_*. Electrical characteristics of the phototransistors, including *μ_sat_*, *I_on_*/*I_off_*, subthreshold swing (SS), and threshold voltage (V_T_) are summarized in Table 1. Compared to the IGZO transistor, the *μ_sat_* and *I_on_*/*I_off_* values of the IGZO/IZO(8:2) transistor were highly enhanced, by about 2.1 and 2.3 times, respectively.

**Figure 2:**
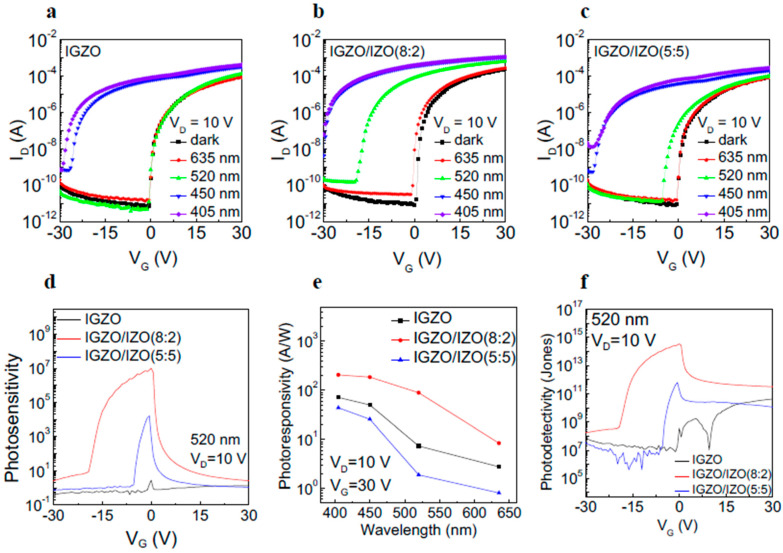
Transfer curves of (**a**) IGZO, (**b**) IGZO/IZO(8:2), and (**c**) IGZO/IZO(5:5) TFTs under various wavelength light illumination (635, 520, 450, and 405 nm). (**d**) Photosensitivity of the IGZO, IGZO/IZO(8:2), and IGZO/IZO(5:5) phototransistors under visible light illumination (λ = 520 nm, P = 4.5 mW cm^−2^) at V_D_ = 10 V. (**e**) Photoresponsivity of the IGZO, IGZO/IZO(8:2), and IGZO/IZO(5:5) phototransistors under illumination with light of various wavelengths at V_D_ = 10 V and V_G_ = 30 V. (**f**) Photodetectivity values of IGZO, IGZO/IZO(8:2), and IGZO/IZO(5:5) phototransistors under wavelength light illumination (λ = 520 nm, P = 4.5 mW cm^−2^) at V_D_ = 10 V.

Figure 2a–c shows the transfer curves of the IGZO, IGZO/IZO(8:2), and IGZO/IZO(5:5) phototransistors under illumination with various wavelength lights (λ = 635, 520, 450, and 405 nm), respectively. As seen in Figure 2a,b, the IGZO phototransistor exhibited photoresponse at wavelengths below 450 nm, while the IGZO/IZO(8:2) phototransistor started to show photoresponse at wavelengths below 520 nm. The IGZO/IZO(5:5) phototransistor also showed slight photoresponse behavior under 520 nm wavelength light illumination. The IGZO/IZO(8:2) device exhibited the most enhanced photoresponse properties under visible light illumination. Figure 2d shows the photosensitivity of the IGZO, IGZO/IZO(8:2), and IGZO/IZO(5:5) phototransistors under 520 nm wavelength light illumination, and it was calculated at V_D_ = 10 V as a function of V_G_ using the following equation:

$$Photosensitivity=\frac{I_{photo}-I_{dark}}{I_{dark}}$$

where *I_photo_* and *I_dark_* indicate the *I*_D_ under illumination and without illumination, respectively [13]. The photosensitivity of the IGZO/IZO(8:2) phototransistor exhibits the highest value at all V_G_ compared to the IGZO and IGZO/IZO(5:5) phototransistors. Furthermore, the photosensitivity gradually increased within the V_G_ range from −30 V in the off state to the turn-on voltage and rapidly decreased in the V_G_ range from the turn-on voltage to 30 V. In the off-state, where V_G_ is smaller than the turn-on voltage, the quantity of photo-excited charge carriers surpasses that of bias-induced charge carriers, resulting in an elevation in photosensitivity. On the other hand, when the devices were turned on, the contribution of bias-induced charge carriers outweighed that of photo-excited charge carriers, leading to a decrease in photosensitivity. Consequently, the variation in the contribution ratio of photo-excited carriers and bias-induced carriers to the channel current, depending on the V_G_, results in a different trend in photosensitivity. Table 2 shows the specific values of photosensitivity at a wavelength of 520 nm for the IGZO, IGZO/IZO(8:2), and IGZO/IZO(5:5) phototransistors. The photosensitivity value of the IGZO/IZO(8:2) device is approximately 10^7^ orders of magnitude higher compared to the IGZO single-layer device. Figure 2e represents the photoresponsivity values of the devices under 635, 520, 450, and 405 nm wavelength light illumination at V_D_ = 10 V and V_G_ = 30 V. The values of photoresponsivity under illumination with wavelengths of 635, 520, 450, and 405 nm with an exposure power of 4.5 mW cm^−2^ were calculated using the following equation:

$$Photoresponsivity=\frac{\left(I_{total}-I_{dark}\right)A_{pt}}{P/A_{pd}}=\frac{J_{ph}}{P}$$

where *I_total_* is the total current in the transistor under visible light illumination, *I_dark_* is the dark current, *P* is the power of the incident light, *A_pt_* is the product of the channel width and thickness, *A_pd_* is the spot size of the laser source, and *J_ph_* is the photocurrent density [37]. Consistent with the photosensitivity values, the photoresponsivity value of the IGZO/IZO(8:2) phototransistor at a wavelength of 520 nm exhibits the highest value at 89.1 AW^−1^. The photodetectivity of the IGZO, IGZO/IZO(8:2), and IGZO/IZO(5:5) devices under 520 nm wavelength light illumination is shown in Figure 2f, using the following equation:

$$Photodetectivity=\frac{\sqrt{A}\cdot R}{\sqrt{2qI_{dark}}}$$

which includes parameters such as channel area (*A*), photoresponsivity (*R*), electronic charge (*q*), and dark current (*I_dark_*) [38]. Among the three devices, the IGZO/IZO(8:2) device manifests the greatest photodetectivity value under 520 nm wavelength light illumination. The noise equivalent power (NEP) for evaluating the noise characteristics of phototransistors is shown in Figure S7. The NEP was calculated using the equation (D* = *√A*/*NEP*), where D* is the photodetectivity under 520 nm wavelength light illumination, and *A* is the channel area [39]. Among the devices, the IGZO/IZO(8:2) device exhibited the lowest NEP value.

**Figure 3:**
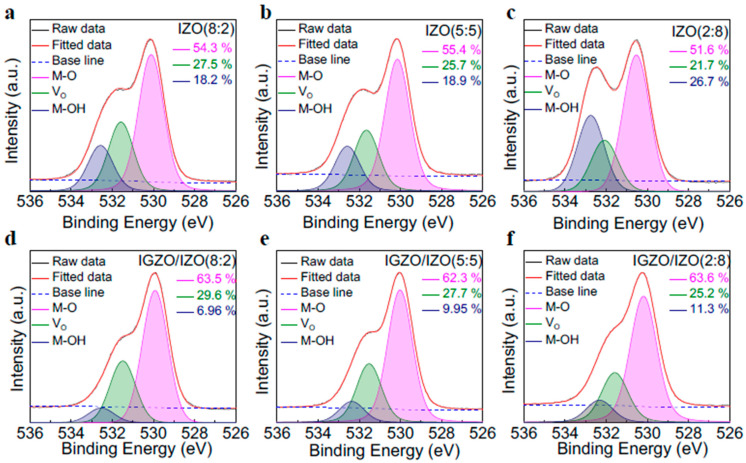
XPS measurement for O 1s of (**a**) IZO(8:2), (**b**) IZO(5:5), and (**c**) IZO(2:8) single layer films and (**d**) IGZO/IZO(8:2), (**e**) IGZO/IZO(5:5), and (**f**) IGZO/IZO(2:8) heterostructure films.

To examine the distinct distribution of In and Zn ratios within the IZO layer, an XPS analysis was performed. The XPS spectra of the indium 3d (In 3d) and zinc 2p (Zn 2p) for IGZO/IZO(8:2), IGZO/IZO(5:5), and IGZO/IZO(2:8) films are shown in Figure S5a,b, respectively. As the ratio of In:Zn changes to 8:2, 5:5, and 2:8, a reduction in In 3d intensity and an increase in Zn 2p intensity were observed. To further investigate the origin of the electrical properties and photoresponse characteristics under visible light wavelength range, the O 1s XPS spectra were measured. Figure 3a–c represents the O 1s spectra of IZO(8:2), IZO(5:5), and IZO(2:8), respectively. Furthermore, to identify if there were any differences in chemical states when the IZO films were deposited above the IGZO film, the O 1s XPS spectra of IGZO/IZO(8:2), IGZO/IZO(5:5), and IGZO/IZO(2:8) were also measured, as shown in Figure 3d–f, respectively. The deconvoluted O 1s peak for the IGZO single-layer film is shown in Figure S6. All the O 1s spectra were deconvoluted into three peaks: metal-lattice oxygen (M-O), oxygen vacancy (V_O_), and metal-OH compounds (M-OH). The peak centered at the binding energy of approximately 530, 531, and 532 eV indicated the M-O, V_O_, and M-OH compounds, respectively [40]. Table 3 summarizes the areal ratio of M-O, V_O_, and M-OH for the IZO single layer and IGZO/IZO heterostructure films. In both the IZO single layer and IGZO/IZO heterostructure films, the amount of oxygen vacancies decreased as the ratio of In:Zn in IZO shifted from 8:2, 5:5, and 2:8. Moreover, compared to the IZO single layer films, the amount of oxygen vacancies increased when the IZO layer was deposited above the IGZO layer. These results arise from the potential increase in interfacial defects by forming the heterostructure [41]. This observation is further supported by the transmittance spectra of IGZO and IGZO/IZO(8:2) films, as shown in Figure 1c. Since the transmittance of the IGZO film is higher than that of the IGZO/IZO(8:2) film, it suggests the presence of an increased amount of oxygen-related states near the valence band in IGZO/IZO(8:2) [42]. Thus, the increased V_O_ was considered to influence the improvements in the optoelectronic properties of the device.

**Figure 4:**
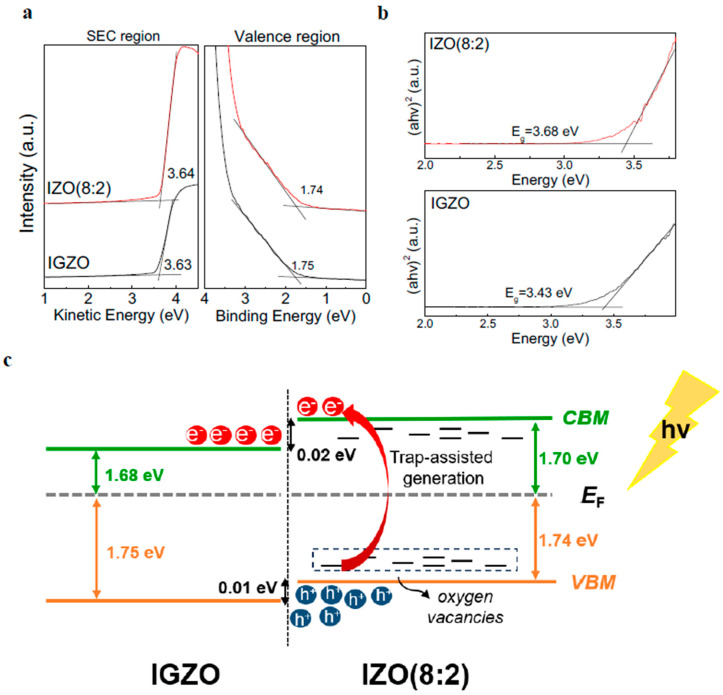
(**a**) UPS spectra of IGZO and IZO(8:2) films in the SEC and valence regions. (**b**) Tauc plots of the IGZO and IZO(8:2) films. (**c**) Schematic interfacial band alignment of IGZO/IZO(8:2) and diagram of photo-generated charge carrier transportation under visible-light wavelength at V_G_ < 0.

UPS measurements were conducted to examine the interfacial electronic band structure of the optimized IGZO/IZO(8:2) structure. Figure 4a shows the secondary electron cutoff (SEC) region and valence region spectra of IGZO and IZO(8:2) films. The work function and valence band maximum (VBM) values were determined from the SEC region and valence region, respectively. The work function values for IGZO and IZO(8:2) are 3.63 and 3.64 eV, respectively. The energy levels between the Fermi level (*E*_F_) and VBM are 1.75 and 1.74 eV for IGZO and IZO(8:2), respectively. The optical band gap (*E*_g_) was obtained from the Tauc plot based on UV–vis spectroscopy analysis, as shown in Figure 4b. The band gap values for IGZO and IZO(8:2) are 3.43 eV and 3.44 eV, respectively. Based on the results from the UPS measurement and Tauc plot, the operating mechanism of the IGZO/IZO(8:2) device under visible light wavelength illumination was schematically illustrated in Figure 4c. The conduction band offset between IGZO and IZO(8:2) was 0.02 eV, and the offset between the valence band was 0.01 eV. Additional states were formed by oxygen vacancies near the VBM region of IZO(8:2), and IZO(8:2) acts as an absorbing layer [30,43]. Therefore, when the IGZO/IZO(8:2) phototransistor was illuminated by visible light wavelength, photo-excited electron–hole pairs were generated under the low photon energy of visible light wavelength. Utilizing trap-assisted generation with oxygen vacancies has improved optoelectronic performance under visible light wavelength light illumination [44]. After photo-generated electrons were excited to the conduction band of IZO(8:2), the electrons immediately transferred to the conduction band of the IGZO layer owing to the internally generated electric field. In contrast, the movement of holes is suppressed due to the potential barrier between IGZO and IZO(8:2) [1,30]. This effective charge separation prevents the recombination of the photo-generated electron–hole pairs, resulting in the improved performance of the phototransistor. Even in the case of the IZO single layer, trap-assisted states can arise owing to oxygen vacancies. However, in contrast with the IGZO/IZO heterostructure, the IZO single layer could exhibit an inferior photocurrent and faster recombination due to the absence of a charge separation layer, as shown in Figure S8. Consequently, these results demonstrate enhanced performance in heterostructure devices compared to single-layer devices. 

**Figure 5:**
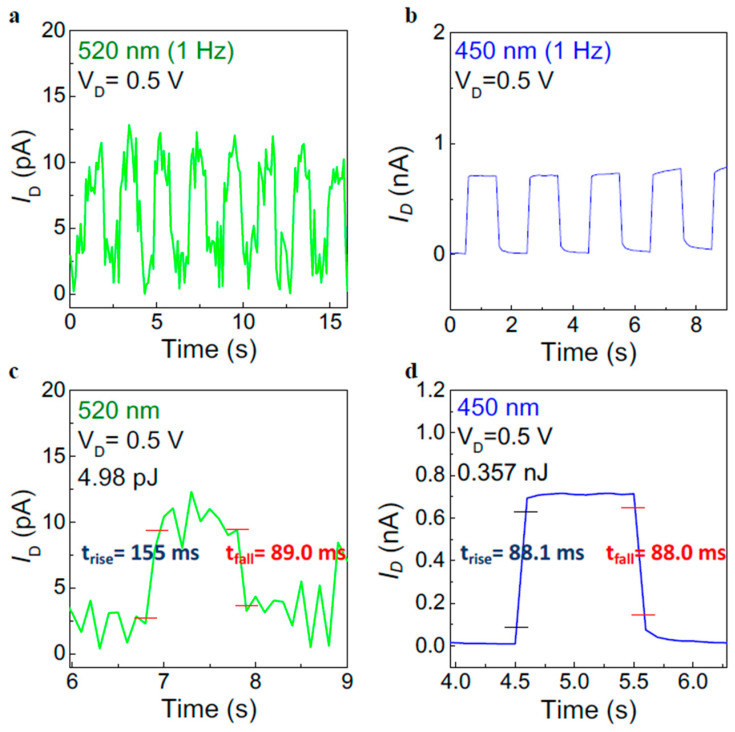
Photoresponse characteristics of the IGZO/IZO(8:2) phototransistor under periodic wavelength light of (**a**) 520 nm and (**b**) 450 nm, and the rising time, falling time, and power consumption of the IGZO/IZO(8:2) at wavelength light (**c**) 520 nm and (**d**) 450 nm. Photoresponse characteristics of the device were conducted under conditions of illumination power = 4.5 mW cm^−2^, V_G_ = −4.5 V, and frequency = 1 Hz.

To further demonstrate the low-power characteristics, we conducted the photoresponse properties of the IGZO/IZO(8:2) phototransistor under periodic light illumination. Figure 5a,b shows the photoresponse properties under illuminating wavelengths of 520 and 450 nm, respectively. The illumination conditions were set at a light power density of 4.5 mW cm^−2^ and a frequency of 1 Hz. The IGZO/IZO(8:2) transistor effectively operates even at a low V_D_ value of 0.5 V without the persistent photocurrent (PPC) effect [23,45]. Moreover, Figure 5c,d shows the rising time, falling time, and power consumption of periodic photoresponse at wavelengths of 520 and 450 nm, respectively. The rising and falling times were defined as the time taken to reach 10% and 90% of the peak photocurrent value, respectively, to verify the low-power operation capability of the phototransistor [46,47]. Under a wavelength of 520 nm, the IGZO/IZO(8:2) TFTs exhibited a rising time of 155 ms and a falling time of 89.0 ms, and under a wavelength of 450 nm, a rising time of 88.1 ms and a falling time of 88.0 ms. The restriction in measurements due to low current levels can result in slight differences between the observed values and actual photoresponse times. Power consumption during the transient operation of the device was calculated using the following equation:

$$E=V_{D}\times I_{D}\times ∆t$$

where *V_D_* is the constant drain voltage of 0.5 V, *I_D_* is the drain current, and ∆*t* is the time pulse of 1 s [48]. The average power consumption was 4.98 pJ at a wavelength of 520 nm, and 0.357 nJ at a wavelength of 450 nm, indicating a low power level. Consequently, the IGZO/IZO(8:2) heterostructure phototransistor exhibited enhanced optoelectronic performance, achieving low power consumption, rapid photoresponse, and minimizing the PPC effect. 

## 4. Conclusions

We have conducted an analysis of the enhanced optoelectronic properties of the oxide-based IGZO/IZO(8:2) heterostructure phototransistor and demonstrated its low power consumption. The *μ_sat_* and *I_on_*/*I_off_* of the IGZO/IZO(8:2) device showed improved electrical characteristics, with a *μ_sat_* of 1.51 cm^2^ V^−1^s^−1^ and *I_on_*/*I_off_* of 3.03 × 10^7^. Additionally, it exhibited superior photoresponse characteristics, including a photosensitivity of 1.00 × 10^7^, photoresponsivity of 89.1 AW^−1^, and photodetectivity of 3.55 × 10^14^ Jones at a visible light wavelength of 520 nm. The enhancement in optoelectronic properties was analyzed through XPS and UPS measurements. The largest amount of oxygen vacancies was observed in the IGZO/IZO(8:2) film, and the performance of the phototransistor was elevated by the trap-assisted generation of oxygen-related states due to oxygen vacancies. Moreover, effective charge separation, resulting from the conduction band offset and valence band offset between the IGZO and IZO(8:2), increased the photocurrent and reduced recombination. With these improved characteristics, the IGZO/IZO(8:2) phototransistor operated with a low power consumption of 4.98 pJ and 0.357 nJ under 520 and 450 nm wavelength light illumination, respectively. These results provide a useful way for fabricating visible-light phototransistors with low power consumption and enhanced optoelectronic performance. Such enhanced-performance phototransistors can be applied to various applications, including image sensors and biometric sensors.

## Figures and Tables

**Table 1:** Summarized *μ_sat_*, *I_on_*/*I_off_*, S/S, and V_T_ values of IGZO, IGZO/IZO(8:2), and IGZO/IZO(5:5) TFTs.

	*μ_sat_* [cm^2^V^−1^s^−1^]	*I_on_*/*I_off_*	S/S [mV·Decade^−1^]	V_T_ [V]
IGZO	0.717	1.31 × 10^7^	389	1.94
IGZO/IZO(8:2)	1.51	3.03 × 10^7^	324	−0.710
IGZO/IZO(5:5)	0.651	5.81 × 10^6^	518	1.85

**Table 2:** Summarized photosensitivity at light wavelength 520 nm and photoresponsivity under various wavelength light illumination (520, 450, and 405 nm) values of the IGZO, IGZO/IZO(8:2), and IGZO/IZO(5:5) phototransistors.

	Photosensitivity (520 nm)	Photoresponsivity [A/W] (520 nm)	Photoresponsivity [A/W] (450 nm)	Photoresponsivity [A/W] (405 nm)
IGZO	2.85	7.34	50.0	71.3
IGZO/IZO(8:2)	1.00 × 10^7^	89.1	185.1	205.1
IGZO/IZO(5:5)	1.66 × 10^4^	1.89	25.7	44.0

**Table 3:** Summarized areal ratio of deconvoluted O 1s XPS spectra of IZO(8:2), IZO(5:5), IZO(2:8), IGZO/IZO(8:2), IGZO/IZO(5:5), and IGZO/IZO(2:8).

	M-O [%]	V_O_ [%]	M-OH [%]
IZO(8:2)	54.3	27.5	18.2
IZO(5:5)	55.4	25.7	18.9
IZO(2:8)	55.6	21.7	26.7
IGZO/IZO(8:2)	61.1	29.6	9.3
IGZO/IZO(5:5)	62.3	29.6	7.0
IGZO/IZO(2:8)	63.6	25.2	11.3

## Data Availability

The authors declare that all data supporting the findings of this study are available within the paper and its Supplementary Information.

## Supplementary Materials

The following supporting information can be downloaded at https://www.mdpi.com/article/10.3390/ma17030677/s1, Figure S1: EDS mapping image; Figure S2: AFM images of IGZO and IGZO/IZO; Figure S3: Transfer curves of IGZO/IZO devices; Figure S4: Transfer curves of IZO devices; Figure S5: XPS spectra of In 3d and Zn 2p; Figure S6: O 1s peak of IGZO film; Figure S7: NEP of IGZO/IZO devices; Figure S8: Schematic diagram of IZO single-layer device operating mechanism.
