# Supplementary material for: Low-Power Phototransistor with Enhanced Visible-Light Photoresponse and Electrical Performances Using an IGZO/IZO Heterostructure
Source: Materials (Basel). 2024 Jan 30;17(3):677. doi: 10.3390/ma17030677 (PMC10856061; doi:10.3390/ma17030677)
Supplement: Supplementary file 1 [file materials-17-00677-s001.zip › materials-2830452-supplementary.pdf]

materials

Article

Low power phototransistor with enhanced visible-light photoresponse and electrical performances using a heterostructure IGZO/IZO

Yu Bin Kim [1], Jun Hyung Jeong [1,2], Min Ho Park [1,2], Jung Min Yun [1], Jin Hyun Ma [1,2], Hyoun Ji Ha [1,2], Seong Jae Kang [1,2] and Seong Jun Kang [1,2,*]

[1] Department of Advanced Materials Engineering for Information and Electronics, Kyung Hee University, Yongin 17104, Republic of Korea
[2] Integrated Education Program for Frontier Materials (BK21 Four), Kyung Hee University, Yongin 17104, Republic of Korea
* Correspondence: junkang@khu.ac.kr; Tel.: +82-31-201-3324

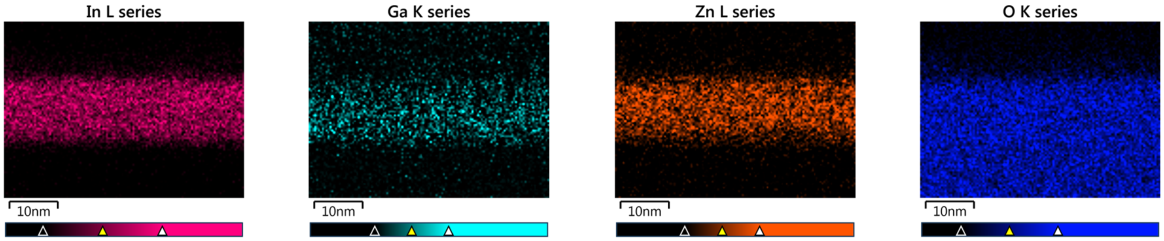

Figure S1. EDS mapping image of In, Ga, Zn and O of IGZO/IZO(8:2) film.

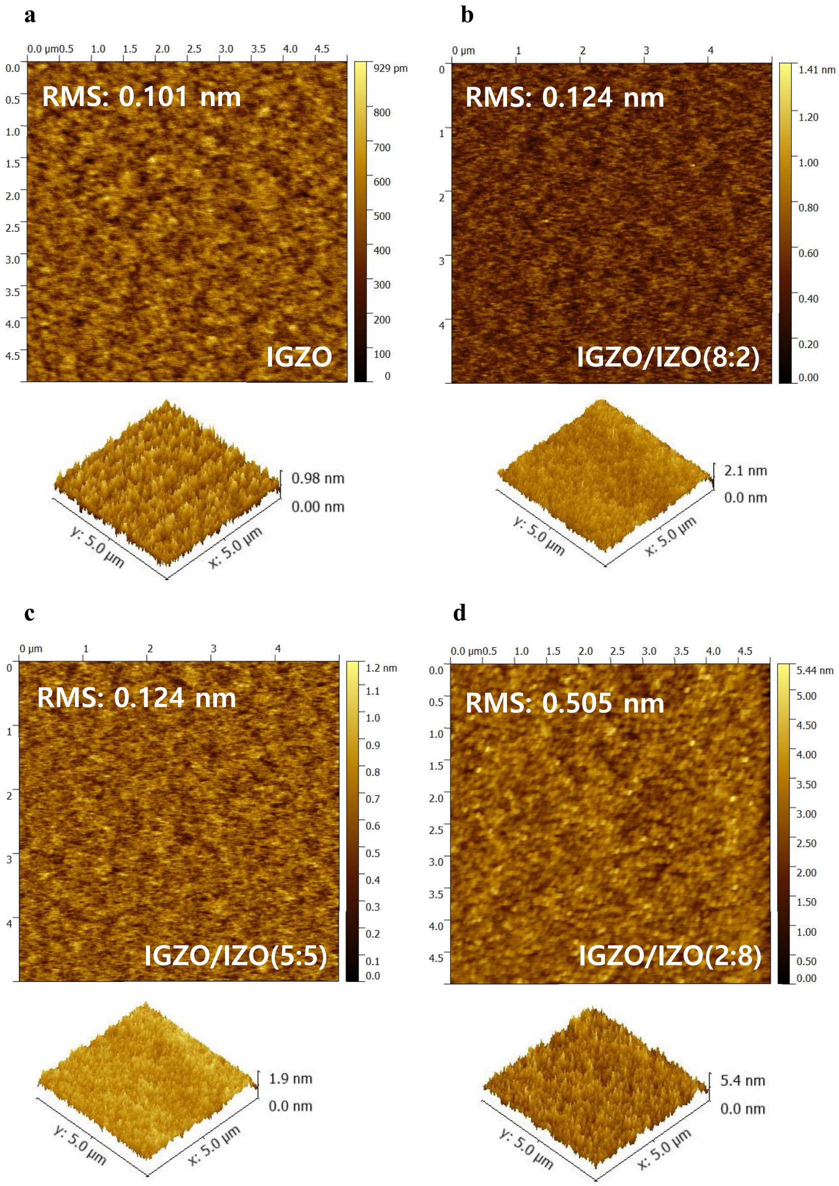

Figure S2. AFM image for 3D topography and rms values ((a). IGZO, (b). IGZO/IZO(8:2), (c). IGZO/IZO(5:5) and (d). IGZO/IZO(2:8) films).

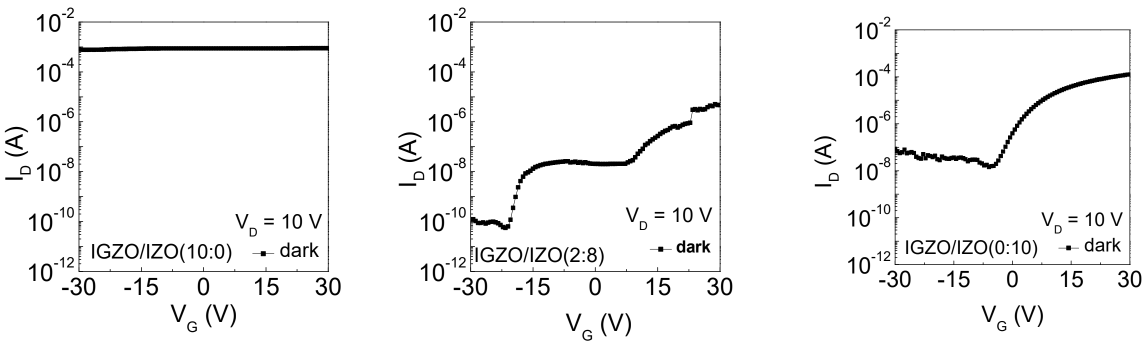

Figure S3. The transfer curves of IGZO/IZO(10:0), IGZO/IZO(2:8) and IGZO/IZO(0:10) heterostructure phototransistors.

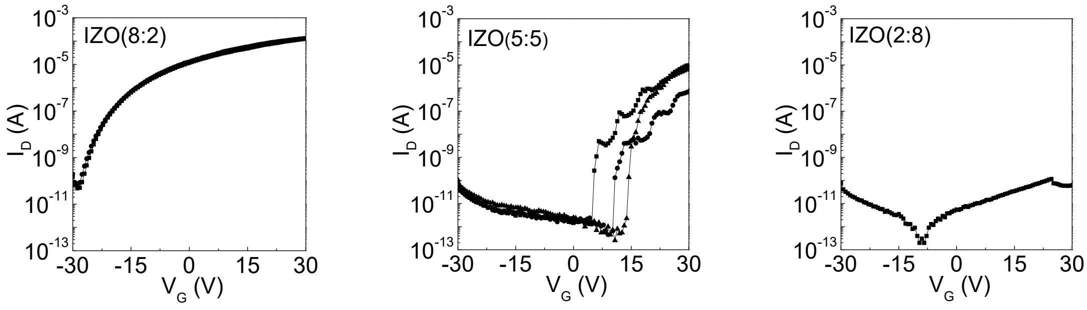

Figure S4. The transfer curve of IZO (8:2), (5:5) and (2:8) single layer phototransistors.

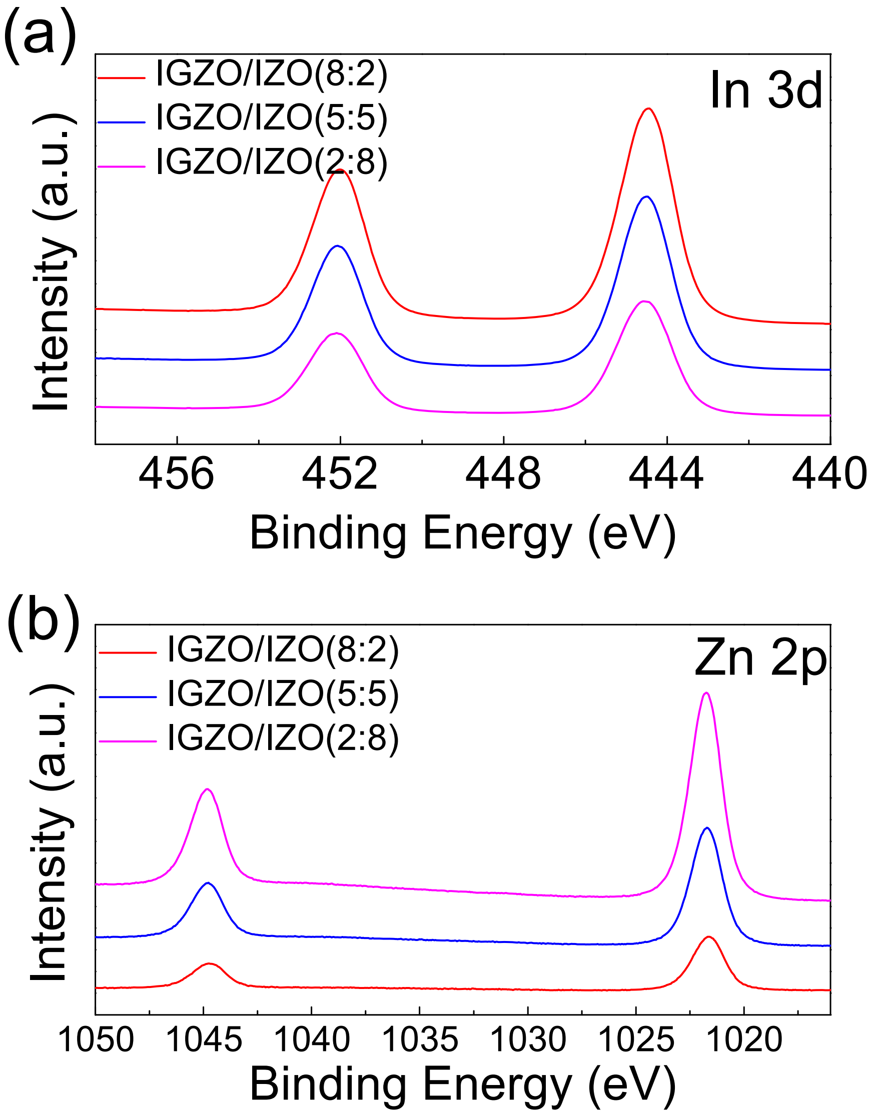

Figure S5. (a) In 3d XPS spectra of IGZO/IZO(8:2), IGZO/IZO(5:5) and IGZO/IZO(2:8) films. (b) Zn 2p XPS spectra of IGZO/IZO(8:2), IGZO/IZO(5:5) and IGZO/IZO(2:8) films.

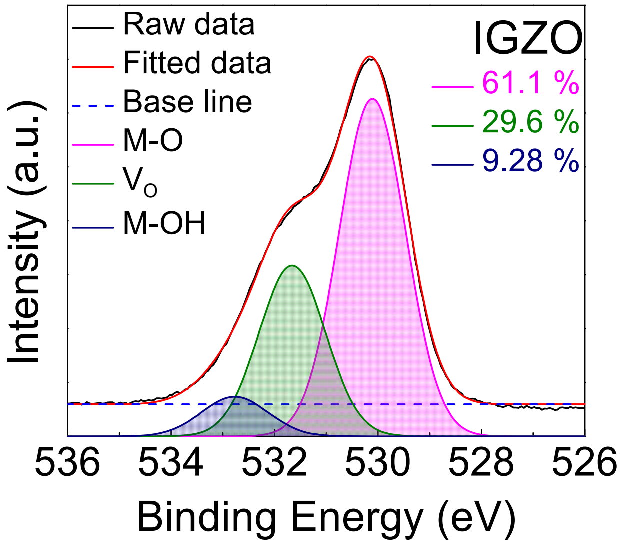

Figure S6. O 1s spectra of IGZO single film.

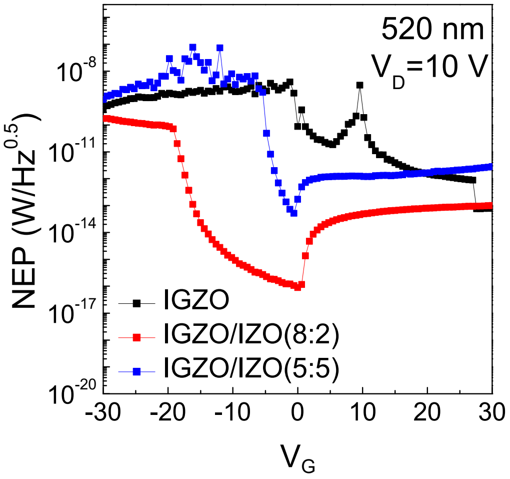

Figure S7. NEP indicating noise characteristics for the IGZO, IGZO/IZO(8:2), IGZO/IZO(5:5) devices.

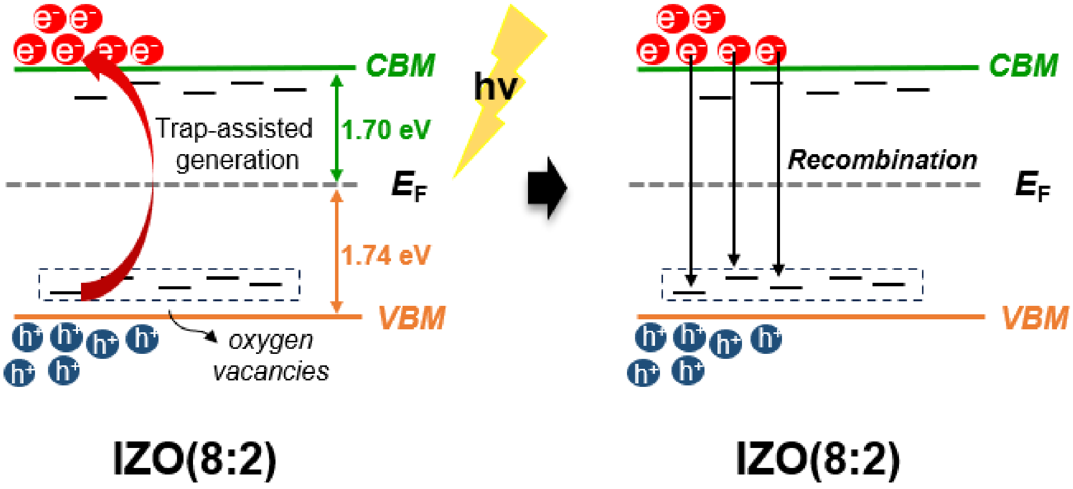

Figure S8. Schematic diagram of photo-excited charge generation and charge recombination in the IZO single layer under visible-light wavelength at $V_G<0$.
